# Case-control association analysis of rheumatoid arthritis with candidate genes using related cases

**DOI:** 10.1186/1753-6561-1-s1-s33

**Published:** 2007-12-18

**Authors:** Yun Joo Yoo, Guimin Gao, Kui Zhang

**Affiliations:** 1 Section on Statistical Genetics, Department of Biostatistics, University of Alabama at Birmingham, 1665 University Boulevard, Ryals Building 327H, Birmingham, Alabama 35294, USA

## Abstract

We performed a case-control association analysis of rheumatoid arthritis (RA) for several candidate genes using the North American Rheumatoid Arthritis Consortium (NARAC) data provided in Genetic Analysis Workshop 15. We conducted the case-control association analysis using all related cases and unrelated controls and compared the results with those from the analysis of samples using only one randomly selected case from each family and all unrelated controls. For both analyses we used a weighted composite likelihood ratio test based on single-nucleotide polymorphism (SNP) markers or haplotypes accounting for the correlation among samples within a family. Several SNPs, including R620W in the candidate gene *PTPN22*, showed an association with RA status, which confirmed previously reported results. Several other SNPs in the candidate genes, such as *CTLA4*, *HAVCR1*, and *SUMO4*, also had rather small *p*-values (<0.05), suggesting the associations between them and RA. Our results showed that the *p*-values obtained from the analysis including all related cases were generally smaller than those obtained from the analysis including only one randomly selected case per family. These results, together with the results, based on simulated data, showed that higher power could be achieved using all related cases.

## Background

Case-control studies using unrelated case and control samples provide a powerful and efficient way to assess the association between candidate genes and diseases. However, case-control studies with related case samples are not uncommon, especially for follow-up studies from a linkage study using pedigrees and/or affected sib pairs. The association analysis based on related cases can be more effective than that based on unrelated cases randomly selected from the population [1] because having more affected members in families will increase the difference in risk allele frequencies between cases and controls [2]. When related cases are available, a simple method for conducting a case-control analysis is to select one case from each family randomly. Although this method is analytically simple, it results in lower power compared with methods that include all related case samples. On the other hand, the use of all related cases without appropriately accounting for their correlations will result in inflated type I error rates. Therefore, many methods were developed to account for the correlation among related cases [1-4]. Browning et al. [3] proposed a weighted composite likelihood ratio test in which the weights of samples are calculated according to their kinship coefficients. One advantage of this method is that it is applicable to both single markers and haplotypes. In this study, we performed an association analysis of rheumatoid arthritis (RA) status with two candidate gene data sets of related cases and unrelated controls from the North American Rheumatoid Arthritis Consortium (NARAC) data provided in Genetic Analysis Workshop 15. We applied the method developed by Browning et al. [3] to these data sets and compared the *p*-values with those obtained from the analysis including only one randomly selected case from each family. We also assessed how the exclusion of candidate variants can affect the analysis and performed the analysis based on multiple markers and haplotypes to identify the effect of multiple single-nucleotide polymorphisms (SNPs).

## Methods

### Weighted composite likelihood ratio test using kinship coefficients

Here, we briefly introduce the weighted composite likelihood ratio (WCLR) test developed by Browning et al. [3]. Denote the individual *i*'s genotype at a single marker as $g_{i}=(g_{i_{1}},g_{i_{2}})$ for *i *= 1, 2,...,*n*, where $g_{i_{1}}$ and $g_{i_{2}}$ are one of the alleles *A*_1_,...,*A*_*l *_with the corresponding frequency $p_{A_{1}},...,p_{A_{l}}$. The weight of each individual is denoted as *w*_*i*_. A weighted composite likelihood of a single marker is then $L(p)=\prod_{i=1}^{n}{\left(p_{g_{i_{1}}}p_{g_{i_{2}}}\right)}^{w_{i}}$, where *p *= ($p_{A_{1}},...,p_{A_{l}}$). Based on this composite likelihood, the allele frequency can be estimated for only cases ($\hat{p}$), only controls ($\hat{q}$), and all cases and controls ($\hat{r}$). A likelihood ratio test can be constructed as $2\ln(L(\hat{p},\hat{q})/L(\hat{r}))$ and it asymptotically follows a chi-square distribution with *l *- 1 degrees of freedom. Similarly, the likelihood ratio test based on haplotypes and a given set of weights can also be constructed. In general, haplotypes for samples are unknown. In this situation, the haplotype frequencies can be estimated via the expectation-maximization (EM) algorithm and incorporated into the test.

The weight *w*_*i *_of each individual is calculated based on their kinship coefficients. Specifically, *w *= (*w*_1_,...,*w*_*n*_) satisfies 2(*w*_1_,...,*w*_*n*_)*K *= (1,...,1), where *K *is the *n *× *n *kinship matrix. The sum of weights, *w*_1 _+ ... + *w*_*n*_, can be considered as the effective sample size, which is greater than the number of families but less than the total number of samples. Thus, we expect the power of this method to be greater than that of the method using one randomly selected case per family. Finally, it is worth noting that *w*_*i *_= 1 for unrelated individuals. In this situation, the weighted composite likelihood becomes the ordinary likelihood.

### Candidate gene analysis

We analyzed two candidate gene data sets from NARAC using CCREL software that implements the WCLR test of Browning et al. [3]. The first data set contains genotypes of 1256 cases from 665 nuclear families and 1519 unrelated controls at 14 SNP markers in the candidate gene *PTPN22 *[5]. The 14 SNPs are labeled as SNP1 to SNP14 corresponding to their positional order along chromosome 1. One family was excluded from the analysis because of genotyping errors. Most of the parents' genotypes were not available. The second data set contains genotypes of 816 cases from 461 nuclear families and 855 unrelated controls at 20 SNP markers in 14 candidate genes, including *PTPN22*, *CTLA4*, *TNFRSF1B*, *PADI4*, *HAVCR1*, *IBD5*, *SLC22A4*, *IL3*, *IL4*, *SUMO4*, *ILG5*, *CARD15*, *RUNX1*, and *MFL *[6]. We performed the case-control association analysis using two methods. In the first method we used all cases and controls and applied the WCLR test. In the second method, we randomly selected one case from each family and included all unrelated controls and applied the allelic chi-square test. For the first data set, we performed the single marker analysis, the multiple marker analysis (the stepwise logistic regression), and the haplotype analysis on two or three markers. We also obtained the linkage disequilibrium (LD) measure (*r*^2^) between SNPs from this data set. For the second data set, we performed the single-marker analysis because this data set only includes a few SNPs for each candidate gene.

### Power comparison

The power of the two methods based on all related cases and all controls, the weighted composite likelihood ratio test [3] and the method suggested by Slager and Schaid [1], and the power of the method using only one randomly selected case per family and all controls were evaluated and compared based on simulated data. One thousand data sets were generated and each of them consisted of 200 affected sib pairs (400 cases) and 200 unrelated controls. Only genotypes at the disease locus were simulated and analyzed. The minor allele frequencies at the disease locus for cases and controls were set as 0.141 and 0.095 to calculate the power, and set as 0.10 and 0.10 to calculate the type I error rate. The significance level was set as 0.05.

## Results

### Association of RA status with *PTPN22*

Table 1 shows the results of the single-marker analysis using all cases and controls and using one randomly selected case per family and all controls for each SNP of *PTPN22 *provided in the first data set. The minor allele frequencies at each marker locus were estimated separately for cases and controls. SNP3, SNP5, SNP7, SNP9, SNP13, and SNP14 had *p*-values less than 0.05 for both methods. For SNP6, SNP10, and SNP12, the analysis including all cases yielded *p*-values less than 0.05 but the analysis including one randomly selected case did not. SNP9 (R620W) showed the strongest association among all 14 SNPs using both methods. Most of the *p*-values for the analysis including all related cases were smaller than those for the analysis including one randomly selected case per family.

**Table 1:** Single-marker analysis for SNPs in PTPN22

		**Minor Allele Frequencies**			***p*-values^a^**	
				
		**Cases**		**Controls**		
**SNP**	**dbSNP ID**	**Affected Sibling^b ^(n = 1256)**	**One Sibling per Family^c ^(n = 664)**	**(n = 1519)**	**Affected Sibling^b^**	**One Sibling per Family^c^**

1	rs3789604	0.198	0.199	0.179	0.10251	0.11214
2^d^	rs3811021	0.199	0.200	0.179	0.0978	0.10504
3	rs1217413	0.269	0.267	0.217	7 × 10^-5^	0.00039
4^d^	Ss38346942	0.015	0.015	0.013	0.68537	0.63067
5	rs1217388	0.292	0.293	0.251	0.00233	0.00436
6	Ss38346943	0.017	0.020	0.027	0.03122	0.13409
7	rs1310182	0.491	0.495	0.430	5 × 10^-5^	7 × 10^-5^
8^d^	Ss38346944	0.03	0.031	0.023	0.19819	0.15854
9^d^	rs2476601	0.154	0.160	0.084	4.32 × 10^-13^	8.53 × 10^-13^
10	rs12730735	0.268	0.270	0.295	0.04785	0.09262
11^d^	rs11102685	0.091	0.085	0.076	0.0629	0.32266
12	rs12760457	0.267	0.269	0.294	0.04884	0.09406
13	rs2488458	0.292	0.293	0.252	0.00257	0.00472
14^d^	rs1217414	0.243	0.236	0.276	0.01283	0.00526

^a^*p*-Values were not adjusted for the multiple testing.

^b^Allele frequencies and *p*-values were obtained using all cases and unrelated controls.

^c^Allele frequencies and *p*-values obtained using one randomly selected case per family and unrelated controls.

^d^SNPs selected from the stepwise regression.

Using the data of one randomly selected case per family, we performed a stepwise logistic regression to see whether the association with SNP9 in *PTPN22 *accounted for the associations were observed with other SNPs. Six SNPs, SNP2, SNP4, SNP8, SNP9, SNP11, and SNP14 were included in the final model according to the Akaike Information Criterion (AIC). Among them, SNP2, SNP4, SNP8, and SNP11 did not show the associations with RA status in the single-marker analysis, suggesting the existence of the epistatic effect between them. SNP3, SNP5, SNP7, and SNP13 were in moderate LD with SNP9 and were not included in the final model, indicating that the associations observed with these SNPs were accounted for by SNP9.

We excluded SNP9, which was reported to have the strongest association with RA status, from the haplotype analysis to see whether the haplotypes of other SNPs could capture its effect. We first performed the haplotype analysis based on sliding windows with two or three SNPs. Several haplotypes showed the associations with RA status but haplotypes formed by two SNPs closest to SNP9 (SNP8 and SNP10, both were in low LD with SNP9) did not. We then performed the haplotype analysis for five SNPs in the stepwise logistic regression model (SNP2, SNP4, SNP8, SNP11, SNP14) and found several haplotypes that showed significant associations with RA status (Table 2). We also performed the haplotype analysis for 3 SNPs that showed moderate LD (*r*^2^:0.3~0.5) with SNP9 (SNP3, SNP5, SNP13) and found that all haplotypes showed significant associations with RA status (Table 2). In the haplotype analysis, most of the *p*-values for the analysis including all cases were smaller than those for the analysis including one randomly selected case per family.

**Table 2:** Haplotype analysis for SNPs in PTPN22

**SNPs selected in the stepwise regression (SNP2, SNP4, SNP8, SNP11, SNP14)**		**SNPs in LD with SNP9 (SNP3, SNP5, SNP13)**	
**Combinations**	***p*-values^a^**	**Combinations**	***p*-values^a^**

2 & 11	0.008	3 & 5	0.0001
2 & 4 & 11	0.016	3 & 13	0.0001
2 & 8 & 11	0.013	5 & 13	0.009
2 & 11 & 14	0.0003	3 & 5 & 13	0.0002

^a^*p*-Values were obtained using all cases and unrelated controls and were not adjusted for the multiple testing.

### Association of RA status with other candidate genes

Table 3 shows the significant results of the single-marker analysis for 20 SNPs in 14 candidate genes for both methods. As expected, *PTPN22 *showed the strongest association with RA status. Also, all SNPs in candidate gene *CTLA4*, *HAVCR1*, and *SUMO4 *had *p*-values less then 0.05 for the analysis including all cases but some of these *p*-values were not significant for the analysis including one randomly selected case per family.

**Table 3:** Single-SNP case-control analysis for SNPs in 14 candidate genes

		**Minor Allele Frequencies**			***p*-values^a^**	
				
		**Cases**		**Controls**		
**Gene**	**dbSNP ID**	**Affected Sibling^b ^(n = 816)**	**One Sibling per Family^c ^(n = 461)**	**(n = 855)**	**Affected Sibling^b^**	**One Sibling per Family^c^**

*PTPN22*	Rs2476601	0.163	0.156	0.084	2.13 × 10^-10^	4.96 × 10^-8^
*CTLA4*	Rs3087243	0.447	0.407	0.447	0.01016	0.04618
*HAVCR1*	Rs6149307	0.17	0.174	0.142	0.03939	0.03193
*HAVCR1*	5509_5511 delCAA	0.195	0.200	0.235	0.01834	0.05172
*SUMO4*	Rs237025	0.517	0.520	0.456	0.00154	0.00202
*SUMO4*	Rs577001	0.391	0.387	0.350	0.02512	0.06162

^a^*p*-Values were not adjusted for the multiple testing. Only significant results are presented in the table.

^b^Allele frequencies and *p*-values were obtained using all cases and unrelated controls.

^c^Allele frequencies and *p*-values were obtained using one randomly selected case per family and unrelated controls.

### Power comparison

Our simulation results showed that the weighted composite likelihood ratio test [3] had a power of 89.4%, which was very similar to the power of the method developed by Slager and Schaid [1] (89.2%). Both of them had higher power than the method using one randomly selected case per affected sib pair and all unrelated controls (79.0%). All three methods showed appropriate type I error rates close to the nominal level of 0.05 (0.047, 0.048, and 0.053, respectively).

## Discussion

Carlton et al. [5] conducted the case-control association analysis using a subset of the first data set of 14 *PTPN22 *SNPs by randomly selecting one affected case from each family. Their reported *p*-values based on single markers are slightly different from our results for SNP10, SNP11, and SNP12, which might be due to the random selection of the set of case samples. Most of SNPs in the second data set were analyzed by Plenge et al. [6], and our results overall are consistent with their findings. Our results, obtained by randomly selecting one case from each family, varied from one selection to another. In practice, there are no guidelines for choosing the best result because the results generally reflect the selection process.

The results from the stepwise logistic regression analysis showed that the effect of several SNPs disappeared when SNP9 was included in the analysis. These SNP were in moderate LD with SNP9 and showed the associations with RA status in the single marker analysis, indicating their associations with RA status may be mainly due to the association of SNP9 with RA status. Several SNPs that were included in the final regression model did not show the associations in the single marker analysis but showed the associations in the haplotype analysis (e.g., SNP2 and SNP11). SNP2 and SNP1 (strong LD with SNP2, *r*^2 ^= 0.997) have been suggested as having the associations with RA status by Carlton et al. [5].

We also performed haplotype analysis based on two or three SNPs after excluding SNP9 from the *PTPN22 *SNP data set. We wanted to see whether the indirect association between RA and these SNPs could be better captured using haplotype analysis. The two SNPs adjacent to SNP9, SNP8 and SNP10, did not show an association with RA in the haplotype analysis; SNP8 showed no association with RA, and SNP10 showed only marginal association with RA in the single-marker analysis. However, haplotypes based on SNPs in moderate LD with SNP9 showed associations with RA status. Thus, the sliding-window approach may not be sufficient to capture the associations for untyped SNPs. In this situation, one can analyze haplotypes based on all combinations of several SNPs but with rapidly increased number of tests. One can also only analyze haplotypes in high LD with untyped SNPs with reduced number of tests, if the LD patterns can be obtained from publicly available data resources (e.g., HapMap project).

## Conclusion

We analyzed the association between several candidate genes and RA status using all related cases and all unrelated controls. Our analysis showed that several SNPs in the candidate gene *PTPN22 *were significantly associated with RA status with possible epistatic effects. Also, SNPs in *CTLA4*, *HAVCR1*, and *SUMO4 *were significantly associated with RA status.

We compared the results of the analysis including all related cases with those of the analysis including only one randomly selected case per family. The *p*-values from the analysis including all cases were generally smaller than those from the analysis including only one randomly selected case per family, suggesting the higher power of the method using all cases. This was confirmed by the power comparison based on simulated data. Thus, we suggest the use of methods that can use all related cases and can correctly account for the correlations among them, such as the weighted composite likelihood method of Browning et al. [3], rather than the methods that use one randomly selected case per family, in order to yield more power and eliminate inconsistency.

## Competing interests

The author(s) declare that they have no competing interests.
